# Behavioral and physiological insights into cross-stage interactions in the desert locust

**DOI:** 10.1038/s41598-025-08853-y

**Published:** 2025-06-27

**Authors:** Benjamin Fürstenau, Vincent O. Nyasembe, Hosea O. Mokaya, Hillary K. Kirwa, Angel Guerrero, Baldwyn Torto

**Affiliations:** 1 Julius Kühn Institute (JKI), Federal Research Centre for Cultivated Plants, Institute for Ecological Chemistry, Plant Analysis and Stored Product Protection, Königin-Luise-Str.19, 14195 Berlin, Germany; 2 International Centre of Insect Physiology and Ecology (ICIPE), Behavioural & Chemical Ecology Department, P.O. Box 30772, Nairobi, GPO – 00100 Kenya; 3 Department of Biological Chemistry, Institute for Advanced Chemistry of Catalonia (IQAC-CSIC), Jordi Girona 18-26, Barcelona, 08034 Spain

**Keywords:** *Schistocerca gregaria*, Synchronous maturation, Pheromone, Phenylacetonitrile, Chemical ecology, Animal behaviour, Animal physiology, Entomology

## Abstract

Gregarious desert locusts produce stage-specific pheromones that facilitate cohesive behavior in juveniles and synchronize maturation and mating in sexually mature adults. During locust outbreaks, merging populations result in cross-stage interactions, yet their impact on locust biology remains poorly understood. This study tested the hypothesis that cross-stage interactions influence juvenile cohesion and physiological traits. Using behavioral assays and gas chromatography-mass spectrometry, we examined short- and long-term interactions between juvenile and adult desert locusts. In short-term (24 h) cage assays, the presence of adults did not significantly affect grouping behavior in gregarious 3rd instar nymphs, as measured by the mean distance between individuals. Likewise, overall, juvenile pheromone emissions, based on previously identified nymphal components, showed no significant differences regardless of adult presence. Cross-stage interactions also had no measurable effect on the development time of 3rd instar nymphs. In contrast, long-term assays showed that 1st instar nymphs grouped with adults matured faster and grew heavier than older nymphal instars and fledglings, and, as mature males, released higher levels of phenylacetonitrile (PAN). Additionally, adult females emerging from these interactions oviposited earlier and laid more eggs than those not exposed to adults as juveniles. These findings indicate that cross-stage interactions impact development uniquely across different gregarious locust stages. Additionally, they offer important insights into desert locust behavior and chemical ecology, which could aid in developing more effective management strategies.

## Introduction

Locusts are unique grasshoppers because they can occur in two phases- a harmless and cryptic solitarious phase and a swarming gregarious phase^1^ which poses a significant threat to crops and forage and food security^2^. Chemical communication plays a key role in gregarious behavior, as demonstrated in various locust species^3^, including the desert locust *Schistocerca gregaria* (Orthoptera: Acrididae)^4-10^ and the migratory locust *Locusta migratoria* (Orthoptera: Acrididae)^11-15^. In *S. gregaria*, chemical communication is stage-specific, with both sexes of nymphs releasing aggregation pheromone and mature males producing an adult aggregation pheromone^16^. Mature male-produced volatiles accelerate maturation in immature adults^17^, while phenylacetonitrile (PAN), the major component, repels conspecific males during mating^18^. In contrast, *L. migratoria* exhibits a simpler, non-stage-specific pheromone communication system^11-14^.

Desert locust outbreaks can be highly destructive, as swarms migrate over vast distances, with an estimated 40–80 million locusts per square kilometer (FAO, 2021). Each locust consumes food equivalent to twice its body weight (about 2 g) daily, totaling approximately 339 g over its lifetime^19^. A single female can lay up to three egg pods, each containing 80–150 eggs, which hatch into wingless nymphs known as hoppers^2^. The hopper phase consists of five molting instars before maturing into pink fledglings and eventually transforming into sexually mature yellow adults^2^.

The most recent major desert locust outbreak occurred in East Africa between 2019 and 2020, triggered by unusual heavy rainfall in Yemen, Somalia, and Eritrea (FAO, 2021, https://www.fao.org/locusts/en/). The infestation spread to neighboring countries such as Kenya, Uganda, Tanzania and Ethiopia, resulting in massive locust amalgamations and new breeding sites. According to the World Bank (2021), economic losses from crop damage, livestock impact, and asset destruction amounted to approximately $8.5 billion in the region (https://www.worldbank.org/en). Aerial spraying of millions of liters of synthetic toxic insecticides—over one million hectares in Ethiopia and Kenya alone—was used to control the outbreak, raising concerns about environmental consequences^20^.

A key feature of gregarious desert locust amalgamations is cross-stage interactions between different populations and stages, which may positively or negatively affect locust biology. From a positive perspective, they may enhance greater genetic diversity and reproductive success, potentially increasing the fitness of the next generation. On the other hand, they may negatively affect locust by disrupting chemical communication and interfere with synchronized behavior and expose younger nymphs to the risk of cannibalism by older individuals during mass movements^21^. Understanding short- and potential long-term cross-stage interactions is essential for improving locust management strategies. Here, we tested the hypothesis that cross-stage interactions between juveniles and adults would influence juvenile cohesive behavior, development and physiology, as measured by pheromone emissions. Using cage assays and chemical analysis based on coupled gas chromatography-mass spectrometry, we examined short- and long-term cross-stage adult interactive effects on locust juveniles. Our results highlight the complexity of gregarious locust biology and areas for future exploration.

## Materials and methods

*Insects.* A gregarious colony of the desert locust *S. gregaria* was obtained from the Insect and Animal Breeding Unit (IABU) of ICIPE (International Centre of Insect Physiology and Ecology, Nairobi, Kenya). Mixed sexes (ca. 100 individuals) were reared in glass-fronted aluminium cages (50 × 50 × 50 cm^3^ in a well-ventilated room (4.5 × 4.5 m^2^ with a duct system that maintained negative pressure. The different developmental stages were kept separately in cages and maintained at 33 ± 2 °C, 60% RH, and a 12:12 L:D photoperiod. Fresh grass and wheat bran were provided to the insects every day.

### Short-term cross-stage interactions

*Effect of grouping 3rd instar nymphs with adult desert locust on nymphal biological traits.* This experiment compared the effect of grouping 3rd instar gregarious desert locust nymphs (*referred to as recipient nymphs*) with mixed sexes of mature adults (*referred to as signal sources*). Recipient nymphs were exposed to visual, tactile and olfactory cues by the adults. An aluminium cage (50 cm x 50 cm x 50 cm), fitted with a sliding glass door in the front, wire gauze on top, bottom and lateral sides, and the back made of aluminium sheath was used. Twenty 3rd instars (2–3 days old) were placed together with twenty mature adults (females = 10 and males = 10; both 10–12 days old) in a controlled environment room (35 ± 2 °C, 70 ± 5% RH) for 24 h. Another twenty 3rd instar nymphs of similar age not grouped with adults for a similar period comprised the control. This was replicated three times. The biological traits compared in both treatment (recipient) and control nymphs included: (a) cohesion as measured by the mean linear distance in millimeters between two nymphs on two different days at 12:00, 13:00 and 15:00 h (day 1), and at 09:00, 10:00 and 11:00 h (day 2) as nymphal locusts are diurnal marchers. Images of nymph groups, either with or without adults, were acquired at these defined time points following a randomized sampling approach, and linear distances were analyzed using IC Measure 2.0 software (https://ic-measure.software.informer.com/2.0/). At each time point, five measurements were taken per cage involving 10 randomly selected nymphs. (b) Pheromone emission was assessed after 24 h of grouping (see collection and analysis of volatiles). (c) Developmental times were monitored and recorded as the nymphs progressed from the 3rd instar stage to mature adults. After volatile collection, recipient and control nymphs were returned to their respective cages and the time to reach the adult stage was measured. All experiments were carried out in triplicate. Pheromone emissions in mature adults were not measured due to the paucity of samples. Most locusts died by the adult stage which prompted us to conduct subsequent experiments with 1st instars in long-term cross-stage interactions.

## Long-term cross-stage interactions

*Effect of grouping 1st instar nymphs with adult desert locust on nymphal development and maturation.* Based on the results from the short-term cage assays with 3rd instar nymphs, we tested the long-term interactive effect of grouping 1st instar nymphs with adults. We used the same aluminium cages and conditions as in the short-term interactive assays with 3rd instars. Ten 2-day-old 1st instar nymphs (*referred to as recipient nymphs*) from the gregarious colony were placed in one of the experimental cages together with ten 7-day-old immature males and ten females (*referred to as signal sources*) (treatment I). We used individuals of both sexes to account for any possible role of adult females as well, since previous work has shown that maturation signals are associated with only mature adult males^6,22,23^. To exclude possible crowding effect, 70 nymphs of the same age were placed in one cage without being grouped with adults (treatment II). These two assays were replicated six times each (*N* = 6; Table 1). In a control experiment, ten 2-day-old 1st instar nymphs as recipients (= treatment I equivalent) were placed in a cage without adults (control 1; treatment III, *N* = 5; Table 1).

**Table 1:** Treatments to study long-term cross-stage interactive effects of the desert locust by measuring development and different maturation responses of 1st instar nymphs grouped with (exposed) or without (unexposed) conspecific adults and of immature adults grouped with or without conspecific nymphs.

Treatment	Recipients (*R*)	Signal source (S)	No. of insects (*R*:S)
I^a^	Nymphs	Adults	10:20
II^b^	Nymphs	---	70:0
III^c^	Nymphs	---	10:0
IV^d^	Adults	Nymphs	20:10
V^e^	Adults	---	20:0

^a^ 2-day-old 1st instar nymphs grouped with immature adults (7-days old; 10 males, 10 females) (*N* = 6).

^b^ 2-day-old 1st instar nymphs (70) grouped without immature adults (*N* = 6).

^c^ 2-day-old 1st instar nymphs (10) grouped without immature adults as *control 1* (treatment I equivalent) (*N* = 5).

^d^ 7-day-old immature adults (10 males and 10 females) grouped with 2-day-old 1st instar nymphs (*N* = 3).

^e^ 7-day-old immature adults (10 males and 10 females) grouped without nymphs as *control 2* (treatment IV equivalent) (*N* = 3).

To measure the influence of newly emerged nymphs on the maturation of immature adults, ten 7-day-old immature males and ten females (*referred to as recipients adults*) were placed in an experimental cage, together with ten 2-day-old 1st instar nymphs (*referred to as signal sources*) (treatment IV). In another cage (= treatment IV equivalent), we placed ten 7-day-old immature males and ten females without nymphs (control 2; treatment V), which was replicated three times (*N* = 3; Table 1). Insects were supplied with fresh wheat seedlings and wheat bran daily. Biological traits monitored included: (a) mean body weight of the entire group of ten individuals from each cage for treatment I and III 1, 7, 13, 16, 19, 22/23 days after eclosion (for treatment II, 10 nymphs were taken from each test cage and weighed on the different days), (b) percentage of fledged nymphs at 26, 29, 32, 35 days after eclosion, (c) titers of PAN released by single males aged 13–14, 19–20, 25–26, 32–33, 39–40 days after fledging, and (d) mean number of egg pods laid by gravid females 23, 26, 29, 32, 35, 38, 41, 44 days after fledging. In this last experiment, only insects from treatments I, III, IV and V were considered to guarantee similar numbers of recipient individuals (Table 1).

To assess oviposition in gravid females, four aluminium oviposition cups (10 cm x 4.0 cm ID) were placed into a false floor close to the sliding door. The oviposition cups were filled with sterilized, 15% moistened sand^24,25^. The space provided by the experimental cage was ample enough for the chosen numbers of test individuals in each treatment (Table 1). Repeated egg laying by individual locusts was not considered in this analysis. Each recipient insect becoming mature was marked on the abdomen with a black marker pen to differentiate it from the signal source insects (Table 1). Dead signal sources were replaced by new ones of the same age and gender.

## Collection of volatiles

*Short-term cross-stage interactions*. Volatiles were collected from recipient and control 3rd instar nymphs exposed (20 each) for 24 h as previously described^7,26^. Briefly, air from a compressed air cylinder was passed through a charcoal filter over locusts contained in a trapping chamber (25 cm long x 3.5 cm ID), and pulled through a pre-cleaned Super Q (30 mg) in glass tubes (6 cm long x 8 mm ID) (ARS Inc., Gainesville, FL, USA) via a vacuum pump at a flow rate of 350 ml/min. This was replicated three times.

*Long-term cross-stage interactions.* Volatiles were collected in each case from individual recipient males exposed to adult males and females during development (nymphs grouped with adults; treatment I), not exposed to adult volatiles (nymphs without adults; treatment II + III), exposed to male and female nymphs (immature adults grouped with nymphs; treatment IV); not exposed to nymphal volatiles (immature adults grouped without nymphs; treatment V) (Table 1).

For each test, volatiles were collected weekly on randomly selected insects from the different cages at 13–14, 19–20, 25–26, 32–33, 39–40 days post-fledging. The tested males were marked on the abdomen and not used for further analyses. Each collection with a different set of individuals was replicated a minimum of three times (*N* = 3–8). All the trapped volatiles were eluted with 200 µL GC grade dichloromethane (DCM) (Sigma-Aldrich Ltd., Milwaukee, WI, USA) and stored at -80 °C until analysis.

## Analysis of volatiles

An aliquot of 1.0 µL of each sample was analyzed on an Agilent 7890A gas chromatograph equipped with a HP-5 MS capillary column (30 m x 0.25 mm ID x 0.25 μm film thickness), and coupled to a 5795C mass spectrometer (Agilent Technologies, Inc., Santa Clara, CA, USA) with helium as carrier gas (1.2 mL/min). The oven temperature was initially set at 35 °C for 5 min., then programmed at 10 °C/min to 280 °C and held at this temperature for 10 min. Spectra were recorded at 70 eV in the electron impact (EI) ionization mode. The juvenile pheromone components hexanal, heptanal, octanal, nonanal, decanal^7^, and fecal-derived components benzaldehyde, phenol and guaiacol^5^, were identified by comparison of their mass spectra with that of authentic standards and with library spectral data (NIST Registry of Mass Spectral Data, 2008). The levels of these volatiles were compared based on their peak areas. In the long-term cross-stage experiment, the amount of the adult male-specific volatile phenylacetonitrile (PAN) was quantified relative to the amount of the internal standard methyl salicylate (600 ng) added to 40 µL of each volatile extract.

## Chemicals

The synthetic standards including hexanal, heptanal, octanal, nonanal, decanal, benzaldehyde, phenol, guaiacol, phenylacetonitrile (PAN), methyl salicylate, and dichloromethane were purchased from Sigma Aldrich (USA). Purity ranged between 95 and 99%.

### Statistical analysis

In the short-term cross-stage cage assays, cohesive behavior was assessed by measuring distance and analyzing the data using analysis of variance (ANOVA), followed by Tukey’s HSD *post-hoc* test at *P* < 0.05, as they were normal distributed. The mean total volatile emissions of previously identified nymphal pheromone components (hexanal, heptanal, octanal, nonanal, decanal, phenol, and guaiacol)^7^ from recipient and control 3rd instar nymphs were compared using a paired *t*-test. Nymphal development times, with and without exposure to adults, were analyzed using ANOVA followed by Tukey’s HSD *post-hoc* test at *P* < 0.05, after performing a normality assessment using the Shapiro-Wilk test. All statistical analyses were conducted in R (version 4.3.2).

In the long-term cross-stage assays, mean body weight data were checked for normal distribution by the Shapiro–Wilk test and for sphericity by the Mauchly test and analyzed by repeated measures ANOVA with time (days after eclosion) as dependent variable and the different treatments (Table 1) as independent variables. After a significant ANOVA for treatment or treatment x time effects was found, means were separated by Fisher’s LSD *post-hoc* test at *P* < 0.05.

Depending on the data distribution, percentages of fledged individuals were analyzed using a generalized linear mixed model (GLMM), using the package lme4 with a binomial error distribution and a logit link function, with treatment as fixed factors and trials as random factors. Time was treated as a continuous predictor, so the results represent model-based (interpolated) estimates. Residual diagnostics were assessed using the performance package. Post-hoc pairwise comparisons between treatment groups and days were performed using Tukey’s honest significant difference (HSD) test of the emmeans package, with *p*-value adjustment for multiple testing using.

For egg deposition data, non-parametric Kruskal-Wallis rank sum tests were used with Dunn’s tests with Bonferroni correction for multiple comparisons as a non-parametric post-hoc test.

For PAN release, the assumptions of normal distribution and equal variances were not met and Kruskal-Wallis rank sum tests followed by Dunn’s *post-hoc* tests with Bonferroni correction for multiple comparisons were applied at *P* < 0.05.

Analyses were conducted with the software package STATISTICA 99 Edition 5.5 (StatSoft Inc, Tulsa, USA) and in R (version 4.4.1) and R Studio (version 2024.04.2).

## Results

### Short-term cross-stage interactions

**Fig. 1:**
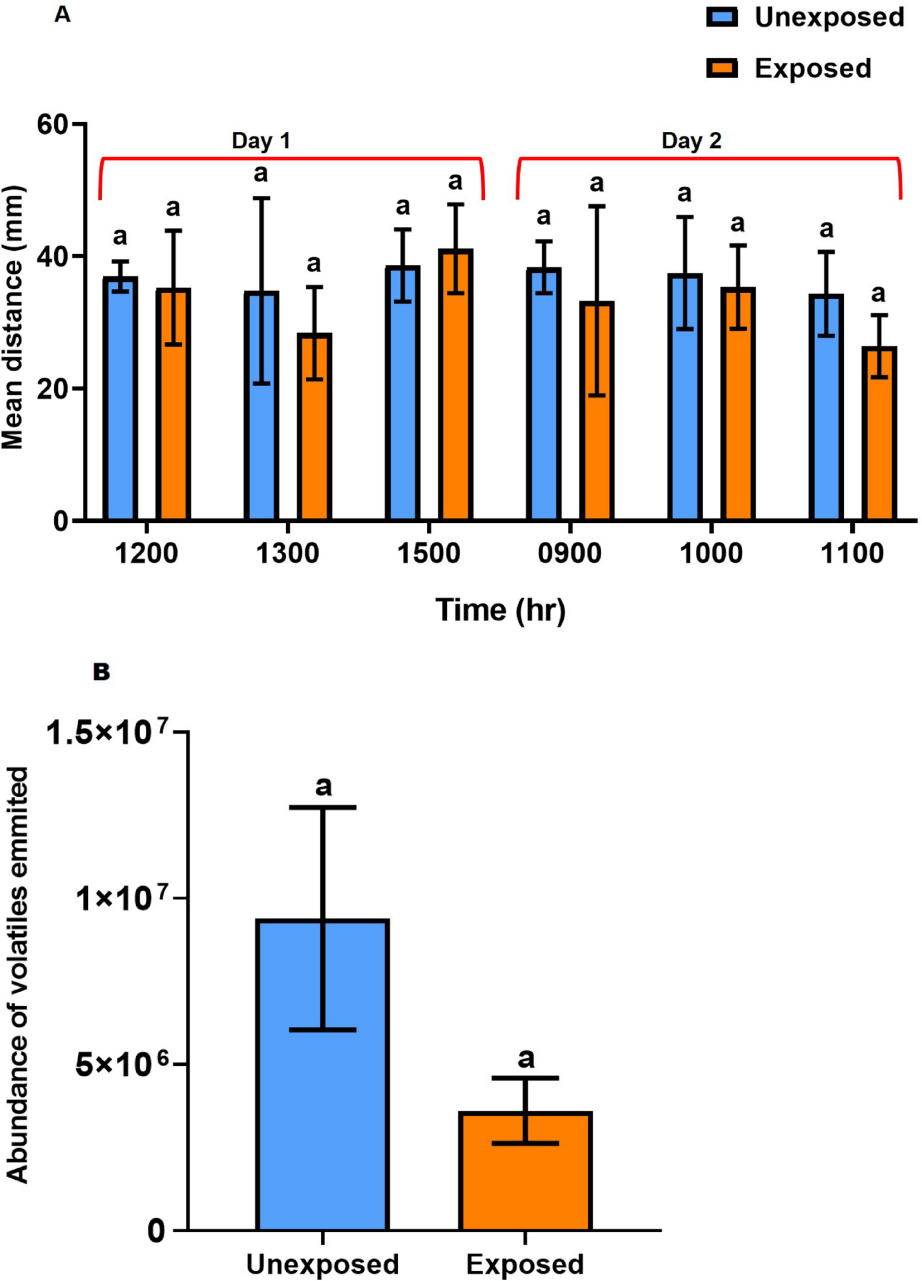

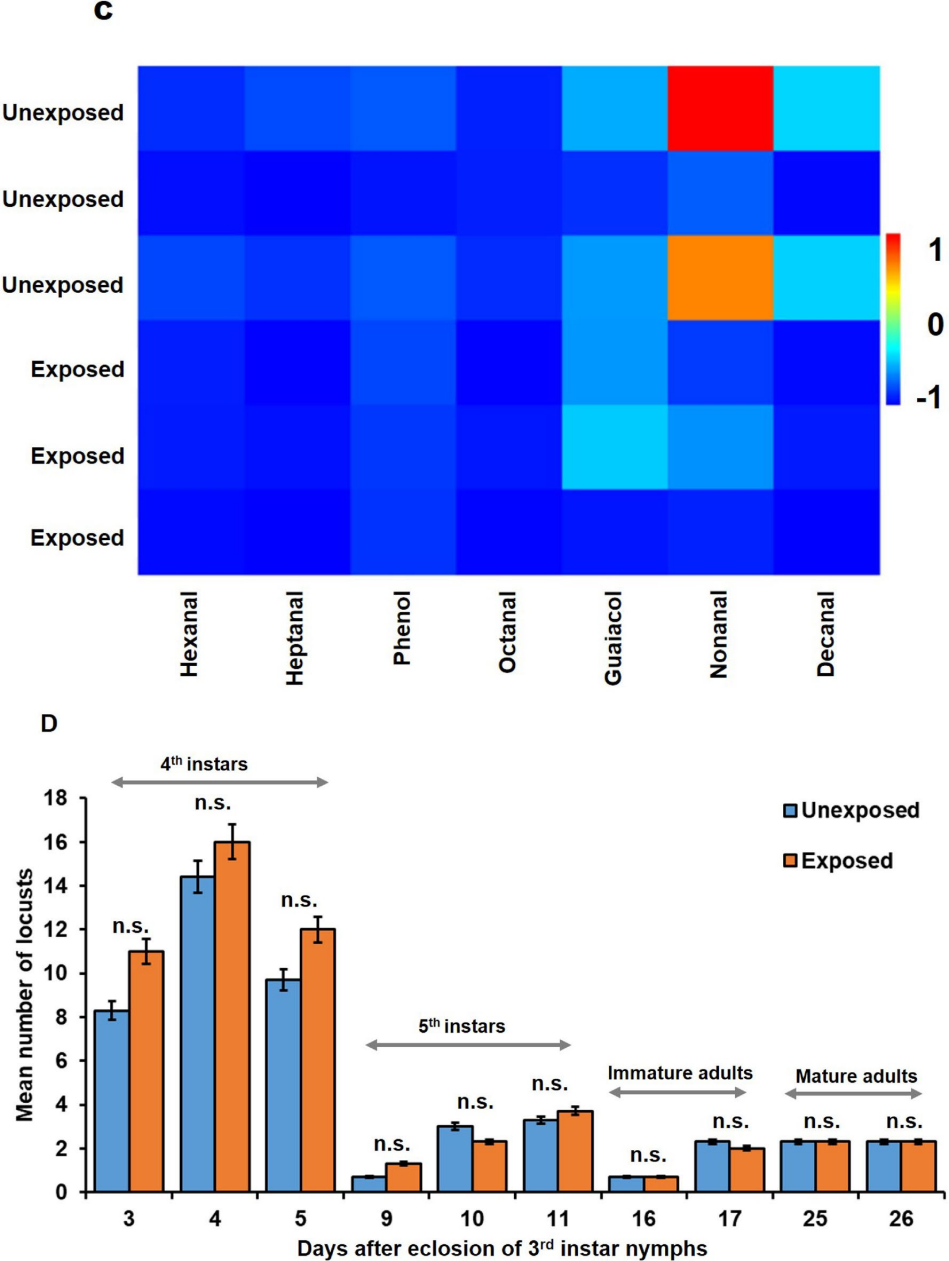
*Short-term cross-stage interactions - Grouping effect on nymphal biological traits of 3rd instar nymphs exposed to adult desert locust*: (A) cohesive behavior measured by nymphal distance (mean ± SE) on two different days at 12:00, 13:00, 15:00 h (day 1), and at 09:00, 10:00, 11:00 h (day 2) (*N* = 15); (B) mean total volatiles emitted (*N* = 3); (C) heatmap of individual volatiles emitted; (D) developmental times of 3rd instar nymphs (20) grouped (exposed) and not grouped (unexposed) with adults (20) of the gregarious desert locust for 24 h (for mean total volatiles emitted, paired *t*-test was used, while One-way ANOVA, Tukey’s HSD test, *P* < 0.05 was used for the other assays). Different letters within a component indicate significant differences between exposed und unexposed nymphs; *n.s.* = not significant).

*Cohesive behavior*. Grouped and ungrouped 3rd instar nymphs with adults did not differ significantly in their cohesive behavior at the random times over the 24 h (*F*_9,20_ = 1.041, *P* = 0.443) (Fig. 1A).

*Pheromone emission*. In short-term (24 h) cage assays, the presence of adults did not significantly affect the mean pheromone emission in gregarious 5th instar nymphs (*t*_1_ = 1.390, *P* = 0.30) (Fig. 1B), although the heat map showed varying levels of specific individual compounds, such as guaiacol, nonanal and decanal between unexposed and exposed nymphs (Fig. 1C). Adult volatiles were not collected in this experiment since most adults died by the time they matured.

*Development time*. The development times for unexposed and exposed 3rd instar nymphs to adults were not significantly different (Fig. 1D): Days after eclosion to 4th instar nymphs- *F*_1,16_ = 2.443, *P* = 0.138, 5th instar nymphs- *F*_1,16_ = 0.021, *P* = 0.886, flegdlings (immature adults)- *F*_1,10_ = 0.05, *P* = 0.828, and mature adults- *F*_1,10_ = 0, *P* = 1.0. After day 7 onwards, most of the test individuals died, but the mean total number of surviving locusts in the unexposed and exposed cages were not significantly different (*F*_1,58_ = 0.267, *P* = 0.607).

### Long-term cross-stage interactions

**Fig. 2:**
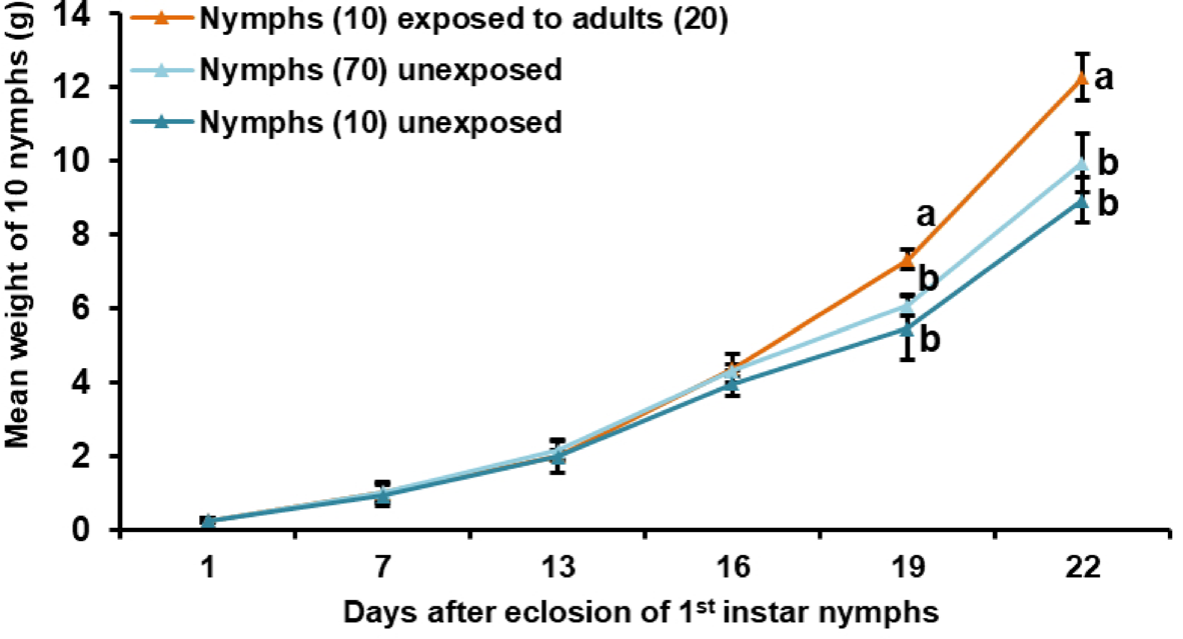
*Long-term cross-stage interactions - Grouping effect on nymphal development of 1st instar nymphs exposed to adult desert locust displayed by body weight*. Increase in body weight of groups of ten nymphs (mean ± SE) over time grouped with 20 adults (I) (exposed), and nymphs grouped without adults (II + III) (unexposed) at different days after eclosion (*N* = 3–6). Mean body weight at different days after eclosion was statistically compared by repeated measures ANOVA. Different letters indicate significant differences among treatments at *P* < 0.05 according to Fisher’s LSD test.

*Development time*. Both treatment (*F*_2_ = 3699.62, *P* = 0.012) and time (days after eclosion) (*F*_5_ = 176.08, *P* < 0.001) had significant effects on mean body weight of groups of ten nymphs. Significant accelerated development displayed by mean body weight was recorded in 1st instar nymphs that were grouped with adults between days 19 and 22 after eclosion compared to nymphs that were grouped without adults (Fig. 2).

Accelerated development displayed by the final molting step from 5th instar nymphs to immature adults occurred in nymphs, which were grouped with adults (treatment I). Treatment (*χ*^*2*^_2_ = 24.28, *P* = 0.006) and time (days after eclosion) (*χ*^*2*^_1_ = 289.95, *P* < 0.001) had significant effects on the mean number of fledged individuals (Table 2). Twenty-three days after eclosion, 27% of recipient nymphs became flegdlings (immature adults), significantly earlier than nymphs that were grouped without adults (treatment II + III) (Table 2). Significant differences in percentages of fledged individuals between treatments were also evident by day 26 post-eclosion, with 73% of molted nymphs grouped with adults (treatment I) compared to 54% (treatment II) and 26% (treatment III) of nymphs grouped without adults. All the nymphs grouped with adults became fledglings by day 29, while nymphs of the other two treatments showed delayed development, reaching 100% of fledged individuals by an additional six days (day 35) (Table 2).

**Table 2:** Final molt from 5th instar nymphs to immature adults (fledglings). Percentages (± SE) of fledged *S. gregaria* nymphs grouped with conspecific adults (treatment I) and without adults (treatment II + III) at different days after eclosion.

	I	Treatment II	III
Days after eclosion	Nymphs (10) with adults (20)	Nymphs (70) without adults	Nymphs (10) without adults
23	27 ± 6^**a**^	6 ± 3^**b**^	0^**b**^
26	73 ± 6^**a**^	54 ± 15^**b**^	25 ± 7^**c**^
29	100^**a**^	89 ± 14^**a**^	60 ± 14^**b**^
32	100^**a**^	97 ± 3^**a**^	85 ± 7^**b**^
35	100^**a**^	100^**a**^	100^**a**^

Mean numbers with different superscript letters in each row refer to significant differences between treatments at *P* < 0.05 according to Tukey’s honest significant difference (HSD) *post-hoc* test after performing a GLMM.

**Fig. 3:**
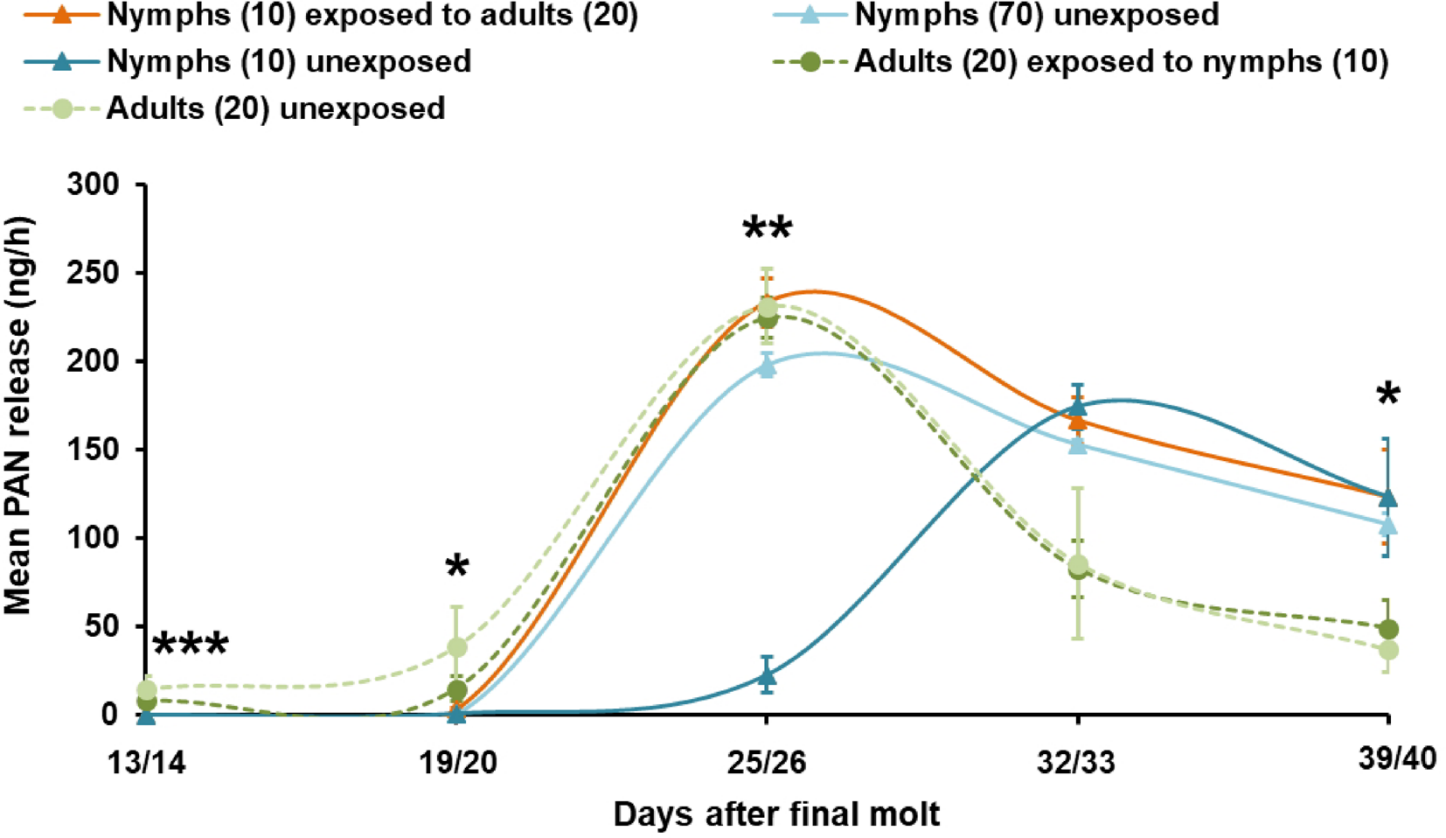
*Long-term cross-stage interactions - Grouping effect on maturation time of male S. gregaria nymphs after final molt displayed by pheromone release*. Emission of phenylacetonitrile (PAN) by single males (mean ± SE) per hour (*N* = 3–8), which during the nymphal phase had been grouped with adults (I), males that were grouped without adults (II + III), and males of the control experiments, which were grouped with nymphs (IV) and without nymphs (V) at different days after fledging. Asterisks indicate significant differences in PAN amount among treatments at different time points (Kruskal-Wallis rank sum tests, *P < 0.05, **P < 0.01, ***P < 0.001).

*Pheromone emission*. Recipient adult males emerged from 1st instar nymphs that were grouped with immature adults started pheromone emission 19–20 days after fledging, releasing 5–8 times higher amounts of PAN per individual (3.4 ± 0.6 ng/h in treatment I) than adult males that emerged from nymphs, which were grouped without adults (0.4 ± 0.4 ng/h in treatment II; 0.7 ± 0.6 ng/h in treatment III) (*H*_4_ = 12.252, *P* = 0.016) (Fig. 3).

Maximum PAN levels were reached earlier by individuals from treatment I and II (25–26 days after fledging) compared to nymphs from treatment III. At this time point, adult males emerged from nymphs grouped with adults emitted significantly more PAN (233.5 ± 13.6 ng/h) than their counterparts that emerged from nymphs grouped without adults (22.6 ± 10.3 ng/h; treatment III) (*H*_4_ = 15.696, *P* = 0.004) (Fig. 3). Males from treatment III released the highest amount of PAN one week later.

Comparison of PAN emission levels of emerged adult males from treatment I-III with those released by emerged recipient adult males from the control treatment IV (immature adults grouped with nymphs) showed significant differences at 13–14 days after final molt (*H*_4_ = 23.449, *P* < 0.001). In both control treatments maximum PAN levels were reached 25–26 days after fledging (224.8 ± 11.55 ng/h in treatment IV; 231.3 ± 20.77 ng/h in treatment V), decreasing rapidly in the days thereafter. On day 32–33, adult males emerged from nymphs that were grouped without adults (treatment III) emitted higher PAN amounts (174.5 ± 12.4 ng/h) than their counterparts that emerged from treatments IV (82.7 ± 16.2 ng/h) and V (85.8 ± 42.8 ng/h), but not significantly different. At 39–40 days after the final molt, no significant differences in the amount of PAN released by adult males were found between the individual treatments (*H*_4_ = 10.491, *P* = 0.033) (Fig. 3).

**Fig. 4:**
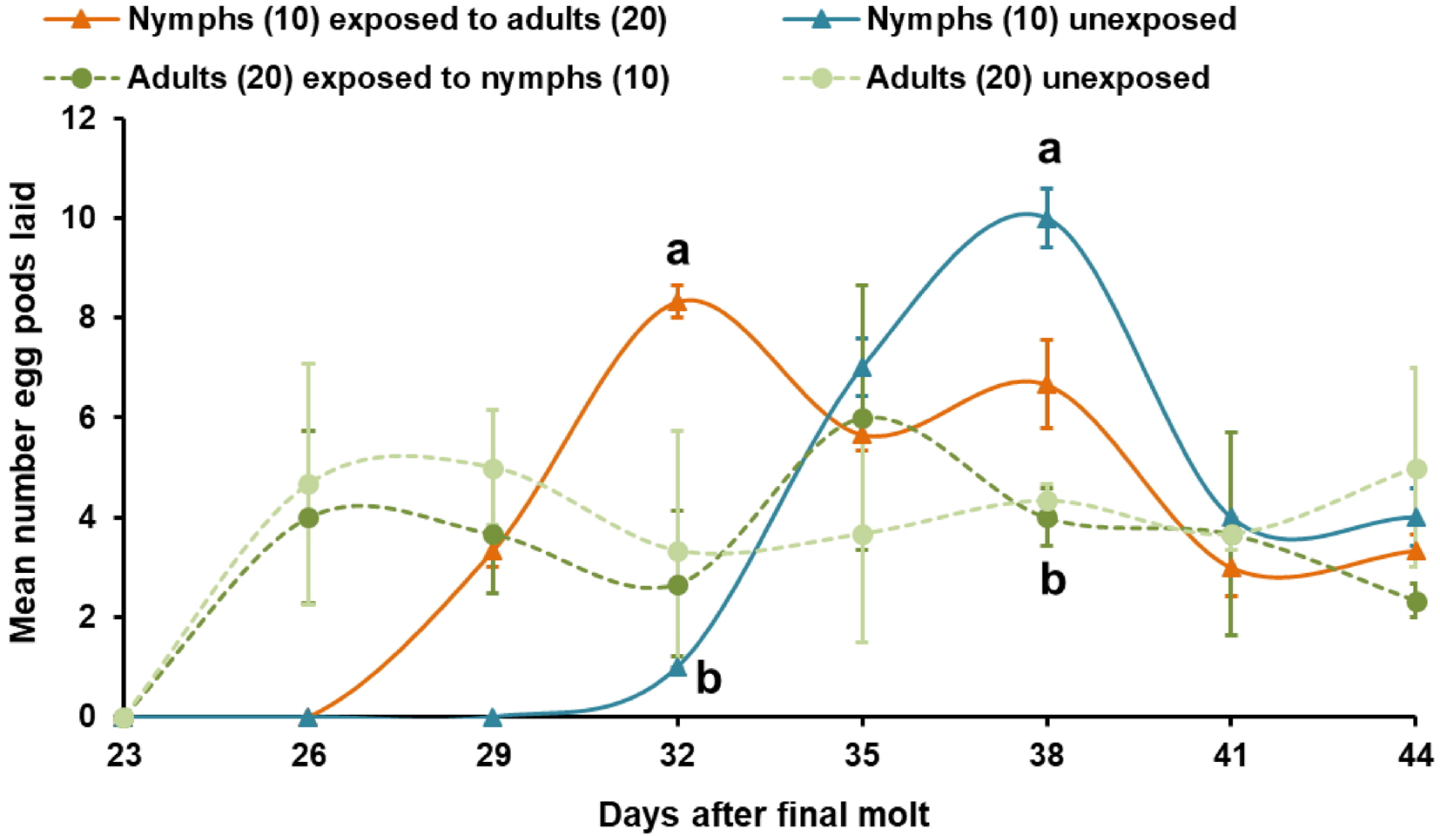
*Long-term cross-stage interactions - Grouping effect on maturation time of female S. gregaria nymphs after final molt displayed by oviposition rate*. Total numbers of egg pods laid by females (mean ± SE), which during the nymphal phase had been grouped with 20 adults (I) (exposed), those which during nymphal phase had been grouped without adults (III) (unexposed) and females of the control experiments which were grouped with nymphs (IV) and without nymphs (V) during the “immature adult phase” (*N* = 3 each). Mean number of egg pods laid was analyzed by Kruskal-Wallis rank sum tests. Different letters indicate significant differences among treatments at specific time points according to Dunn’s *post-hoc* tests with Bonferroni correction for multiple comparisons at *P* < 0.05.

*Oviposition*. A similar maturation pattern was observed in exposed nymphs that emerged as adult females. These females started egg laying three days earlier (day 29) than those emerged from nymphs grouped without adults in treatment III (day 32) (Fig. 4). Emerged adult females from exposed nymphs laid the maximum number of egg pods and significantly more than their unexposed counterparts 32 days after the final molt (*H*_1_ = 4.500, *P* = 0.033) (Fig. 4). Unexposed nymphs reached their egg laying maximum one week later (day 38), laying significantly more egg pods than females emerged from immature adults grouped with nymphs (*H*_3_ = 8.995, *P* = 0.029). Adult females emerged from immature adults exposed and unexposed laid the maximum number of egg pods on day 29 (treatment V) and on day 35 (treatment IV). However, the mean number of egg pods laid was not significantly different from treatment I (Fig. 4). Likewise, the total mean numbers of oviposited egg pods were not significantly different among all treatments (*N* = 3 each): I) 30.3 ± 1.5; III) 26.0 ± 0.6; IV) 26.3 ± 1.3; V) 29.7 ± 6.9 egg pods.

## Discussion

In gregarious desert locusts, both releaser and primer pheromones regulate grouping and maturation behavior^16^. Additionally, tactile cues contribute to gregarious behavior^27^. Our cage assay data indicate that grouping behavior in 3rd instar nymphs remained unchanged regardless of adult presence, as measured by cohesive behavior and pheromone emission^7^. The variations in individual pheromone compound levels are not yet fully understood but may be linked to physiological states, such as the feeding status of individual unexposed and exposed nymphs before pheromone collection. This suggests that under natural short-term locust amalgamations, although nymphs may adjust individual pheromone component emissions, their overall behavioral and physiological responses to adult cues remain stable, potentially optimizing their inclusive fitness.

Mature adult males release PAN, which repels different locust stages^18^. However, our short-term assays suggest that this compound does not significantly interfere with 3rd instar nymphal cohesive behavior or pheromone emission over short exposure periods. A previous study showed that PAN-treated nymphs become disoriented, disperse, and exhibit cannibalistic behavior^16^, a response that appears to differ from interactions with live adults. While we did not assess adult responses to nymphal cues, future work should consider using larger arenas, larger groups, longer exposure times, and different nymphal instar stages while examining responses from both nymphs and adults.

In our long-term cross-stage interactions study, 1st instar nymphs exposed to adults fed more and grew larger as older instars than their unexposed counterparts. This suggests a potential primer effect of adult cues on early-stage nymphs, a response not observed in 3rd instars. However, we cannot rule out density-dependent effects, as the density of 1st instars in this experiment was more than three times higher than that of 3rd instars in the short-term cross-stage assays. Interestingly, a previous study found that 4th instar nymphs of the grasshopper species *Ageneotettix deorum* (Orthoptera: Acrididae) developed faster and grew larger when fed nutritionally superior food in the absence of predation risk from wolf spiders^28^. However, our findings in gregarious locusts revealed the opposite effect- nymphs exposed to potential predation risk developed more rapidly. These observations suggest that grasshoppers, particularly those capable of phase change, may respond differently to intra- and interspecific interactions compared to other grasshopper species. Whether nymphal responses to adult cues in this study were driven by predation or cannibalism risk remains unknown, but the role of sensory-mediated effects in cross-stage interactions warrants further investigation.

Fledglings exposed to mature adult stimuli released higher levels of PAN as mature adult males than those that developed without adult exposure. This finding aligns with previous studies, reinforcing the crucial role that adult sensory cues play in desert locust maturation^6,29^. Under natural conditions, fledglings are more likely to benefit from maturing adult olfactory cues. For example, male-produced 4-vinylanisole has been shown to promote aggregation in the migratory locust *Locusta migratoria* and accelerate maturation in adult females^12,13,14^, although its role in *S. gregaria* remains unclear^10^. Olfactory cues evidently play a significant role in locust behavior. In the Central American locust, *Schistocerca piceifrons*, and the American grasshopper, *S. americana*, (*Z*)-3-nonen-1-ol, released by gregarious mature males, serves as a pheromone that mediates cryptic female mate choice in both locust species^30,31^. Additionally, this compound mediates mate-guarding and induction of sexual maturation in *S. americana*^31^, highlighting the importance of species-specific compounds in locust biology.

Additionally, adult females that emerged from nymphs grouped with adults became gravid one week earlier and laid significantly more egg pods than their unexposed counterparts. This suggests that female nymphs that survive to adulthood respond positively to mature adult male cues, further confirming the role of adult sensory stimuli in gregarious locust maturation^29^.

In summary, our study demonstrates that nymphal responses to adult cues in cross-stage interactions vary depending on the nymphal stage and duration of exposure. These variations appear to influence key biological traits such as morphology, maturation, and pheromone emission, which may enhance nymphal survival during locust amalgamations. Our findings contribute to the understanding of the complex behavior and chemical ecology of the desert locust, providing insights that may inform future management strategies. We recommend further studies on mixed nymphal instars and adults in cross-stage interactions, tracking behavioral and physiological responses throughout their life span.

## Data Availability

The datasets generated and/or analyzed during the current study are available from the corresponding authors on reasonable request.
